# Identification and Complete Genome Sequence Analysis of a Genotype XIV Newcastle Disease Virus from Nigeria

**DOI:** 10.1128/genomeA.01581-15

**Published:** 2016-01-28

**Authors:** Ismaila Shittu, Poonam Sharma, Jeremy D. Volkening, Ponman Solomon, Lanre K. Sulaiman, Tony M. Joannis, Dawn Williams-Coplin, Patti J. Miller, Kiril M. Dimitrov, Claudio L. Afonso

**Affiliations:** a Regional Laboratory for Animal Influenza and Transboundary Animal Diseases, National Veterinary Research Institute, Vom, Nigeria; b Exotic and Emerging Avian Disease Research Unit, Southeast Poultry Research Laboratory, Agricultural Research Service, USDA, Athens, Georgia, USA; c BASE2BIO, Oshkosh, Wisconsin, USA

## Abstract

The first complete genome sequence of a strain of Newcastle disease virus (NDV) from genotype XIV is reported here. Strain duck/Nigeria/NG-695/KG.LOM.11-16/2009 was isolated from an apparently healthy domestic duck from a live bird market in Kogi State, Nigeria, in 2009. This strain is classified as a member of subgenotype XIVb of class II.

## GENOME ANNOUNCEMENT

Virulent strains of avian paramyxovirus serotype 1 (APMV-1) belong to the family *Paramyxoviridae*, subfamily *Paramyxovirinae*, and genus *Avulavirus* (1), and their infections cause Newcastle disease (ND) in birds. APMV-1 (synonymous to Newcastle disease virus [NDV]) is a pathogen capable of causing a devastating disease in domestic fowl, with vast social and economic consequences (2). NDV is an enveloped virus and has a nonsegmented and negative-sense RNA genome with six transcriptional units (3ˊ-NP-P-M-F-HN-L-5ˊ). Phylogenetic analyses of isolates of the virus worldwide have identified large genetic diversity, and currently there are at least 19 different genotypes divided into two major groups (classes I and II) (3, 4). Genotype XIV was recently identified (3) and consequently divided into subgenotypes (4). Viruses of this genotype have predominantly been isolated from rural and commercial gallinaceous poultry in Nigeria, except for two isolates from neighboring Niger (2006) and Benin (2009) (5).

A velogenic NDV (duck/Nigeria/NG-695/KG.LOM.11-16/2009) isolated from an apparently healthy domestic duck from a live bird market in Kogi State, Nigeria, in 2009 was submitted to the Southeast Poultry Research Laboratory of the USDA in Athens, GA. The virus was propagated in 9-day-old specific-pathogen-free embryonated chicken eggs and further processed by next-generation sequencing (NGS). Viral RNA was isolated from the allantoic fluids using the QIAamp RNA viral minikit (Qiagen, USA). NDV RNA capture and enrichment were done using three biotin-labeled oligonucleotide probes using Sera-Mega beads. Reverse transcription was performed using the Moloney murine leukemia virus reverse transcriptase kit (Thermo Scientific, USA). The cDNA products were purified, tagmented, and amplified into Illumina libraries employing the Nextera XT DNA library preparation kit (Illumina, San Diego, CA). Fragment size distribution and concentration of the DNA libraries were checked on a Bioanalyzer 2100 using Agilent high-sensitivity DNA kit (Agilent Technologies, Germany). Paired-end sequencing (2 × 250 bp) of the generated libraries was performed on an Illumina MiSeq instrument using the 500-cycle MiSeq reagent kit version 2 (Illumina). Sequence data were assembled using MIRA version 3.4.0 (6) within a customized workflow on the Galaxy platform (7).

The complete genome length of the isolated strain is 15,192 nucleotides. Comparative analyses revealed that no NDV genomes with high genetic identity (>90%) were available on GenBank (5, 8). Genetic classification was done based on phylogenetic analysis of the complete coding sequence of the fusion protein gene, as previously described (3), and this strain was designated a member of class II, genotype XIV, sub-genotype XIVb.

The strain duck/Nigeria/NG-695/KG.LOM.11-16/2009 contains only basic amino acid residues between positions 113 and 116 of the fusion protein cleavage sites and a phenylalanine at position 117 (_113_RRKR↓F_117_). Such a motif in the deduced amino acid sequence of the cleavage site is specific for virulent NDV isolates (9). The data presented here provide further knowledge on APMV-1 genetic diversity.

### Nucleotide sequence accession number.

The complete genome sequence of NDV strain duck/Nigeria/NG-695/KG.LOM.11-16/2009 has been deposited in GenBank under the accession number KT948996.
